# Inhibition of Nitric Oxide Synthase by L-NAME Promotes Cisplatin-Induced Nephrotoxicity in Male Rats

**DOI:** 10.1155/2013/242345

**Published:** 2013-09-17

**Authors:** Fatemeh Moslemi, Mehdi Nematbakhsh, Fatemeh Eshraghi-Jazi, Ardeshir Talebi, Hamid Nasri, Farzaneh Ashrafi, Maryam Moeini, Azam Mansouri, Zahra Pezeshki

**Affiliations:** ^1^Water & Electrolytes Research Center, Isfahan University of Medical Sciences, Isfahan 81745, Iran; ^2^Department of Physiology, Isfahan University of Medical Sciences, Isfahan 81745, Iran; ^3^Isfahan-MN Institute of Basic & Applied Sciences Research, Isfahan 81546, Iran; ^4^Department of Clinical Pathology, Isfahan University of Medical Sciences, Isfahan 81745, Iran; ^5^Department of Internal Medicine, Isfahan University of Medical Sciences, Isfahan 81745, Iran

## Abstract

*Objective*. Nitric oxide (NO) has numerous important functions in the kidney. The role of NO in cisplatin (CP)-induced nephrotoxicity is not completely understood. This study was designed to determine the role of NO synthase inhibitor (L-NAME) on the severity of CP-induced nephrotoxicity in rats. *Methods*. Sixty four male (M) and female (F) Wistar rats were randomly divided into eight groups. The sham groups (group 1, male, *n* = 6 and group 2, female, *n* = 6) received saline. Groups 3 (male, *n* = 8) and 4 (female, *n* = 8) were treated with L-NAME (4 mg/kg, i.p.), and groups 5 (male, *n* = 8) and 6 (female, *n* = 8) received CP (3 mg/kg) for 7 days. Groups 7 (male, *n* = 8) and 8 (female, *n* = 8) were treated with L-NAME and CP for 7 days. *Results*. The CP-alone treated rats showed weight loss and increase in serum levels of blood urea nitrogen (BUN) and creatinine (Cr). Coadministration of L-NAME and CP did not improve weight loss, and it increased the levels of BUN and Cr in male but not in female rats (*P* < 0.05). CP alone increased kidney damage significantly (*P* < 0.05 ), however, the damage induced by combination of CP and L-NAME was gender-related. *Conclusion*. NOS inhibition by L-NAME increased CP-induced nephrotoxicity, which was gender-related.

## 1. Introduction

Cisplatin (CP) is an effective antineoplastic agent against solid tumors in clinic [1]. Despite the antineoplastic efficacy, the optimal clinical usefulness of CP is usually limited due to its side effects such as nephrotoxicity. CP exerts its nephrotoxic effect mainly in the proximal tubular cells where it is preferentially accumulated [2]. 

The CP-induced nephrotoxicity may be gender related [3], but the mechanism underlying this sex difference is not understood. Studies have shown that female animals are more sensitive to induce acute renal failure (ARF) [4] and CP enhances urinary sodium excretion in male rats but not in females [5]. Different metabolism and sex hormones [6] may involve in CP-induced nephrotoxicity to be sex related.

Nitric oxide (NO) has been suggested to play an important role in CP-induced nephrotoxicity [7, 8]. L-arginine, the precursor of NO, attenuates CP-induced nephrotoxicity in male rats, but it intensifies renal damage induced by CP in female rats [9]. In addition, administration of the nonselective inhibitor of NO synthase, N-nitro L-arginine methyl ester hydrochloride (L-NAME), exacerbates CP-induced nephrotoxicity [10]. The present study was carried out to determine whether administration of L-NAME against nephrotoxicity induced by CP differs between male and female in animal model.

## 2. Methods and Materials

### 2.1. Animals

Sixty-four adult male and female Wistar rats, weighting 176.23 ± 2.15 and 180.77 ± 2.3 g, respectively, (Animal Center, Isfahan University of Medical Sciences, Isfahan, Iran) were used in this research project. The animals were individually housed at a temperature range of 23–25°C. The rats had free access to water and rat chow. The rats were acclimatized to the diet for at least one week prior to the experiment. The experimental procedures were in advance approved by the Isfahan University of Medical Science Ethics Committee.

### 2.2. Experimental Protocol

The animals were randomly divided into eight groups and treated for seven consecutive days as follows.

The sham groups (group 1, male, *n* = 6, and group 2, female, *n* = 6) received saline. Groups 3 (male, *n* = 8) and 4 (female, *n* = 8) received L-NAME (4 mg/kg/day, i.p.), groups 5 (male, *n* = 8) and 6 (female, *n* = 8) received CP (3 mg/kg/day, i.p.), and groups 7 (male, *n* = 8) and 8 (female, *n* = 8) received combination of L-NAME (4 mg/Kg/day, i.p.) and CP (3 mg/Kg/day, i.p.).

The body weight of animals was recorded daily. At the end of the experiment on day 8, all animals were anesthetized with ketamine (75 mg/kg). Blood samples were withdrawn by heart puncture, centrifuged at 6000 rpm for 20 min, and the serum was separated. The serum samples were stored at −20°C until measurement. Then, all animals were sacrificed. The kidneys were removed, freed from the surrounding fats, and weighted immediately. For each animal, the left kidney was fixed in 10% formalin for histopathological investigations. 

### 2.3. Measurements

The levels of serum creatinine (Cr) and blood urea nitrogen (BUN) were determined using quantitative diagnostic kits (Pars Azmoon, Tehran, Iran). The serum level of nitrite (NO stable metabolite) was measured by an ELISA kit that involves the Griess reaction. Briefly, after adding sulfanilamide solution and incubation, N-(1-naphthyl) ethylenediamine dihydrochloride solution was added. Then absorbance was measured with a microreader, and the nitrite concentration of samples was determined by comparison with the nitrite standard reference curve.

### 2.4. Histopathological Procedures

The removed left kidneys were fixed in 10% neutral formalin solution and embedded in paraffin for histopathological staining. The samples were stained by hematoxylin and eosin (H&E) and then were examined for tubular damage by the pathologist. The kidney tissue damage was considered based on the presence of tubular dilation and simplification, tubular cell swelling and necrosis, tubular casts, and intraluminal cell debris with infiltration of inflammatory cells. Based on the intensity of tubular lesions, we scored from 1 to 4, while zero was assigned to the normal tissue without damage.

### 2.5. Statistical Analysis

The data are expressed as mean ± SEM. Statistical analysis of the serum levels of BUN, Cr, nitrite, and percentage of changes in body weight among the groups was performed using the one-way analysis of variance (ANOVA) followed by the least significant difference (LSD) test (post hoc multiple comparison). To compare the pathological damage score between the groups, Kruskal-Wallis and Mann-Whitney tests were used. The *P* value < 0.05 was considered statistically significant.

## 3. Results

From a total number of 64 animals, finally 45 rats survived up to the end of the experiment (Table 1).

### 3.1. Effect of CP on Body Weight

Weight loss induced by CP was expressed as the percentage (%) of body weight change. All CP-treated male and female animals significantly lost weight during the experiment compared with the sham or L-NAME-alone-treated groups. 

**Figure 1:**
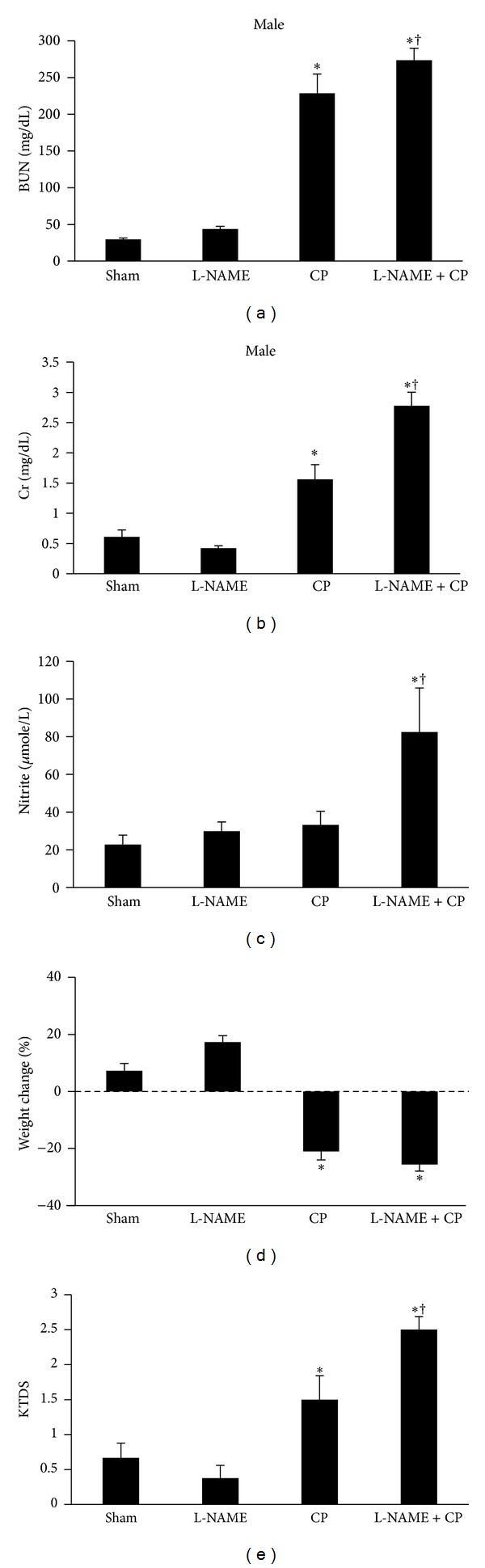
Blood urea nitrogen (BUN), creatinine (Cr), nitrite, percentage of weight change, and kidney tissue damage score (KTDS) in four male groups of sham, treated with L-NAME, CP, and L-NAME + CP. The star and cross symbols indicate significant difference from sham or CP groups, respectively.

L-NAME accompanied with CP did not improve the weight loss induced by CP in both genders. The percentage of body weight change was not significantly different between the L-NAME-alone-treated and sham groups (Figure 1). 

### 3.2. Effect of CP on Serum Levels of BUN and Cr

**Figure 2:**
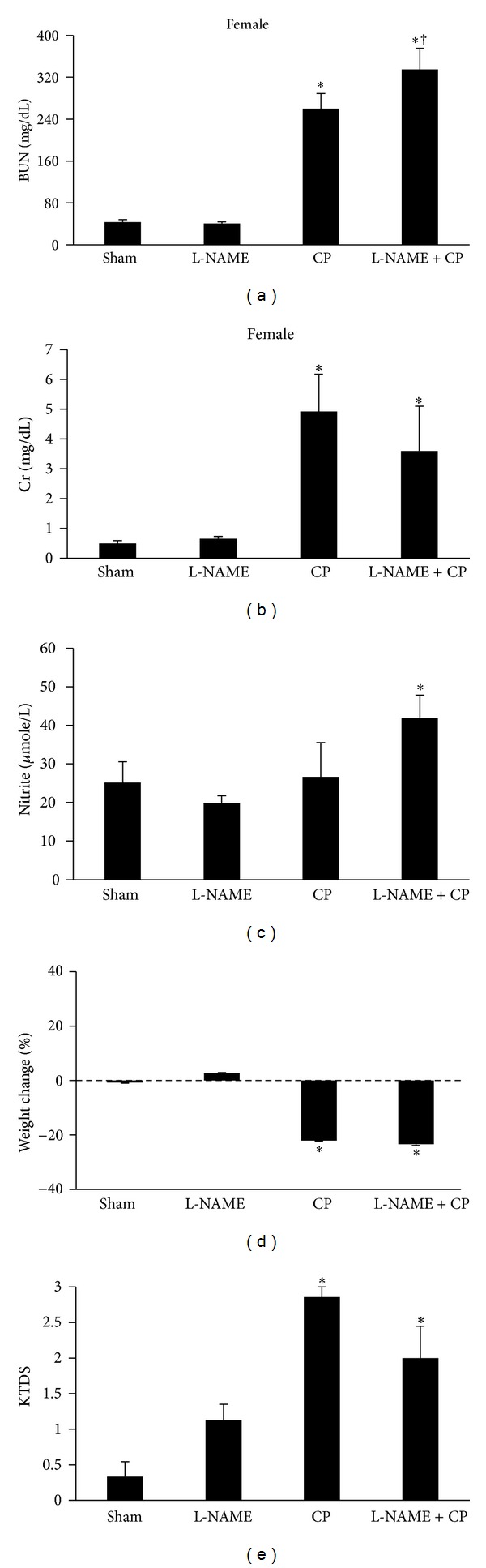
Blood urea nitrogen (BUN), creatinine (Cr), nitrite, percentage of weight change, and kidney tissue damage score (KTDS) in four female groups of sham, treated with L-NAME, CP, and L-NAME + CP. The star and cross symbols indicate significant difference from sham or CP groups, respectively.

The serum levels of BUN and Cr significantly increased in both male and female CP-alone-treated groups in comparison with the sham or L-NAME groups (*P* < 0.05). This was also the case in the L-NAME + CP groups for both genders. The combination of CP and L-NAME elevated the serum levels of BUN and Cr in male when compared with the corresponding CP-alone-treated group, while such finding was not observed for female (Figures 1 and 2).

### 3.3. Effect of CP on Kidney Tissue Damage

The kidney damage induced by CP was evaluated and scored by two independent pathologists. The kidney tissue in the sham and L-NAME groups was considered as normal in two genders. The data showed that the kidney tissue damage induced by CP or by combination of CP and L-NAME significantly increased in male and female rats when compared with the sham or L-NAME-alone-treated groups (*P* < 0.05). However, coadministration of CP with L-NAME significantly enhanced kidney tissue damage in male but not female when compared with the CP-alone-treated groups (*P* < 0.05) (Figures 1 and 2).

**Figure 3:**
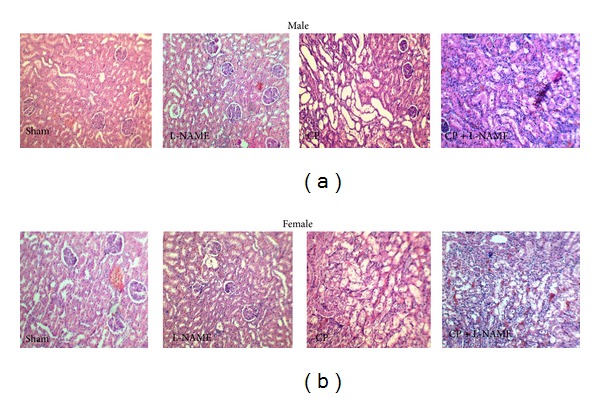
The images of kidney tissues (magnification ×100) in all experiment groups. More kidney tissue damages were observed in groups treated with CP alone or combination of CP and L-NAME.

The images of kidney tissues in all experiment groups is demostraed in Figure 3. More kidney tissue damages were observed in groups treated with CP alone or combination of CP and L-NAME.

## 4. Discussion

CP nephrotoxicity is very complex and includes several mechanisms such as accumulation of the drug in renal epithelial cells, attack of the drug to nuclear and mitochondrial DNA, and initiation of severe inflammatory response [11]. In the present study, we attempted to determine the role of L-NAME on CP-induced nephrotoxicity in male and female rats. 

CP induced significant decrease in body weight probably due to intestinal disturbance [12, 13]; however, the percentage of weight loss was not different between the genders, and L-NAME did not ameliorate the CP-induced weight loss. It is previously reported that pretreatment with L-NAME, as an NO inhibitor, markedly reduces the gastrointestinal toxicity induced by CP [14], and on the contrary, L-arginine, as an exogenous NO donor, increases the CP-induced weigh loss in female gender [15]. However, in this study, it seems that CP was responsible for such weight change. 

Continuous administration of CP increased serum levels of BUN and Cr in both genders at different levels. We obtained the same results in our previous study [3]. In addition, other investigations reported sex-related differences in toxicity induced by CP [15–18], but the mechanism of such sex difference is poorly understood. One parameter, which influences the BUN level, is the body water content. We did not measure the amount of food and water intake. However, CP-induced intestinal disturbance [12, 13] may disturb body water content and serum BUN level.

Accompanying of L-NAME with CP aggravated renal dysfunction in male and female rats by increasing serum levels of BUN and Cr while these makers elevated more in male than female. Our results are in agreement with the results obtained in our previous study. Increasing the amounts of NO by the NO donor (L-arginine) exhibited different effects in male and female [15]. It is documented that L-arginine improves renal failure induced by CP in male [10, 15]. It seems that administration of L-NAME, as an NO inhibitor, aggravates CP-induced renal damage in male but not in female. The possible mechanism for such result may relate to gender dependency of the NO system. Our pathological investigation verified this effect, but, in contrast, Mansour et al. [19] reported that inhibition of all types of NOS with a nonselective inhibitor (L-NAME) or aminoguanidine reduced nephrotoxicity. It is reported that administration of L-NAME results in severe hypertension and causes kidney damage in rats [20]. NO production is also reported to be low in chronic kidney disease (CKD) patients, and NO deficiency may play a role in CKD progression [21, 22]. Chirino et al. [23] demonstrated that excessive NO derived from iNOS after CP administration contributes to the pathogenesis of CP-induced nephrotoxicity and NO has potential beneficial (preservation of renal blood flow and glomerular filtration) and detrimental (cytotoxicity caused by oxidative damage) effects [24]. NO inhibition by administration of nonselective inhibitors results in development of systemic and glomerular hypertension and glomerulosclerosis [20, 25, 26] and may aggravate the development of cyclosporine nephrotoxicity [27] and contrast-induced acute renal failure [28]. The inhibition of NO by L-NAME blocked all sources of NO produced from iNOS, nNOS, or eNOS, and therefore the nonbeneficial effect against CP-induced nephrotoxicity was expected. NO production by CP is also reported to be gender related [29]. We reported before that CP increases the serum total nitrite and nitrite levels in rats [30] and vitamin E could prevent the CP-induced nephrotoxicity via reduction of nitrite or total nitrite [29] gender dependently. It has also been shown that up to 70–90% of plasma nitrite is derived from eNOS activity in fasted subjects [31], while L-NAME inhibits all types of NO including eNOS. Therefore, no protection against CP-induced nephrotoxicity may occur.

It is concluded that administration of L-NAME probably intensifies renal failure induced by CP in male gender. Of course more researches are needed to determine inhibition of which source of NO; iNOS, eNOS, or nNOS, is or is not efficient against CP-induced nephrotoxicity.

## Figures and Tables

**Table 1:** Mortality rate of animals in each group.

Group	Gender	*N*	Day							*n*
			1	2	3	4	5	6	7	
Sham	M	8	—	—	—	—	—	—	—	8
	F	8	—	—	—	—	—	—	—	8
L-NAME	M	8	—	—	—	—	—	—	—	8
	F	8	—	—	—	—	—	—	—	8
CP	M	8	—	—	—	—	—	—	3	5
	F	8	—	—	—	—	—	—	5	3
CP + L-NAME	M	8	—	—	—	—	—	—	4	4
	F	8	—	—	—	—	1	2	2	3

*N*: total number of animals; *n*: number of animals survived up to the end of the experiment; CP: cisplatin; L-NAME: N-nitro L-arginine methyl ester; M: male; F: female.
